# Supplementary material for: Effectiveness and Cost-Effectiveness of Mental Health Interventions Delivered by Frontline Health Care Workers in Emergency Health Services: A Systematic Review and Meta-Analysis
Source: Int J Environ Res Public Health. 2022 Nov 28;19(23):15847. doi: 10.3390/ijerph192315847 (PMC9736335; doi:10.3390/ijerph192315847)
Supplement: Supplementary file 1 [file ijerph-19-15847-s001.zip › ijerph-1935805-supplementary.pdf]

Supplementary: Five categories of key words

Category 1	Category 2	Category 3	Category 4	Category 5
Surviv$	disaster	PFA	health outcome	evaluation
affected	emergenc$	psychological first aid		trial
victim	catastrophe	mental		controlled
		psychology		comparison
				random$
				training

Surviv$ captures survive, survivor, and survivors; emergenc$ captures emergency and emergencies; random$ captures random, randomize, and randomization.
